# Policy options for extending standardized tobacco packaging

**DOI:** 10.2471/BLT.16.190082

**Published:** 2017-08-28

**Authors:** Janet Hoek, Philip Gendall

**Affiliations:** a University of Otago, PO Box 56, Dunedin, Otago, New Zeeland.

Smoking is a public health problem that will cause 1 billion deaths in the 21st century, if current smoking patterns persist.^1^ Tobacco companies’ aggressive marketing has fostered tobacco use by creating brands and linking these to attributes and identities that users and susceptible non-users value. Although many countries now restrict traditional marketing media, tobacco companies have developed increasingly creative strategies to encourage experimentation with tobacco among non-users and deter quitting among users. Packaging has provided an important and until recently, largely unregulated, conduit for these marketing messages and has communicated appealing imagery to young people while using known and familiar brand symbols to reassure existing tobacco users.

The World Health Organization’s Framework Convention on Tobacco Control (FCTC)^2^ obliges Parties to address tobacco packaging. Implementation guidelines for Articles 11^3^ and 13^4^ include consideration of plain packaging and recognize the myriad ways in which tobacco companies have used packaging to promote tobacco use. In response, Australia, France, Georgia, Ireland, New Zealand, Norway and the United Kingdom of Great Britain and Northern Ireland have either introduced or will soon be introducing standardized or plain packaging. Hungary, Romania, Slovenia and Thailand have already passed legislation to introduce plain packaging, and several other countries (including Belgium, Canada, Chile, Singapore and Sri Lanka, among others) are formally considering this policy.

Plain packaging replaces the attractive brand livery displayed on tobacco packs with an aversive colour and standardized text, and is typically combined with larger pictorial warning labels, if these are not already in place. The policy recognizes that on-pack brand imagery has positioned tobacco use as an aspirational behaviour and fostered uptake among young people,^5^ transforming tobacco packages from attractive display items to highly unappealing articles. Evidence from Australia shows standardized packaging and larger pictorial warning labels have reduced smoking’s appeal among young people,^6^ increased demand for cessation support^7^ and decreased smoking prevalence.^8^ Evaluations confirm predictions from experimental and other studies used to support the introduction of this policy.^8^

Despite the intuitive logic of using packaging to deter rather than facilitate experimentation with tobacco products, other countries have been cautious in following Australia’s approach. The time taken for plain packaging to be more widely adopted and implemented in other countries has caused some frustration among public health researchers and advocates. Nevertheless, this time lag has also provided an opportunity to examine how tobacco companies have reacted to Australia’s new policy environment, and to consider whether and where Australian legislation could be extended. We suggest that countries planning to introduce standardized packaging have an opportunity to innovate in at least four ways: (i) restricting or banning brand variant names; (ii) developing larger and more salient on-pack warnings; (iii) requiring tobacco products themselves to use dissuasive colours; and (iv) using packaging to create cessation portals that direct tobacco users to quit support.

First, the introduction of standardized packaging in Australia saw a rapid increase in brand variant names (or descriptors) such as Marlboro Silver Fine Scent, Winfield Optimum Crush Sky and Peter Stuyvesant New York Blend.^9^ These variant names attempt to recreate connotations formerly aroused by visual brand imagery and aim to reassure smokers, deter quitting and potentially attract new users. Recent work suggests the more descriptors used, the more attractive a pack appears.^10^ Variants potentially mislead users by linking tobacco to appealing attributes or by minimizing the harm caused by tobacco use and serve no purpose other than to create marketing appeals.

Given that tobacco cannot be consumed safely, standardized packaging could either preclude the introduction of any new variant names or ban variant names altogether. This former measure would maintain the status quo and be less susceptible to legal challenge, while the latter approach would more assertively prevent packaging from deceiving tobacco users. Alternatively, if reducing tobacco brands to a single presentation (i.e. preventing brands from using different variants as Uruguay has done successfully, despite a legal challenge from Philip Morris)^11^ was congruent with other countries’ legal framework, this approach would constitute an even stronger response to covert tobacco marketing.

Second, plain packaging offers an opportunity to refresh and revise on-pack warnings, thus ensuring that the warning messages and images used will communicate effectively with diverse subgroups within the overall population of tobacco users. Currently, on-pack warnings typically feature diseases caused by tobacco use, such as lung cancer, stroke or cardiovascular disease. Yet while highly relevant to long-term tobacco users who may be experiencing symptoms related to the diseases shown, these warnings lack salience to younger users who may instead view the information provided as irrelevant and exaggerated.^12^ Given that the prevalence of daily tobacco smoking varies substantially across countries by age, sex and ethnicity,^13^^,^^14^ pictorial warning labels should be designed to resonate with groups at greatest risk of future harm. Regulations should thus allow for diverse warnings that illustrate the social as well as physical risks of tobacco use, expose unscrupulous tobacco industry practices, and depict harms that tobacco use inflicts on innocent third parties. Such warning messages have strong effects on young adults and pregnant women, which many countries regard as high priority groups for reducing the harm caused by tobacco use.

Regardless of the content of warning labels, the images’ effectiveness diminishes over time, which is problematic as many countries have been slow to refresh pictorial warning labels. When implementing plain packaging, policy-makers could develop on-going evaluation programmes that regularly test and revise existing warnings, and create mechanisms to fast-track the introduction of new warnings as research evidence supporting these accumulates. Warning development and evaluation programmes should include refreshment schedules that recognize how pictorial warnings’ effectiveness is likely to diminish over time. Regular rotation or replacement of pictorial warning labels will help maintain impact and ensure tobacco users receive varied cessation cues that have the greatest probability of triggering quit attempts.

As part of its plain packaging policy the Australian government increased the size of front-of-pack warnings, which initially covered 30% of the surface, but now take up over 70% of the front surface area. While countries, such as Uruguay, have introduced larger warnings (80% of both the front and back pack areas must feature pictorial warnings) independently of plain packaging, countries developing plain packaging regulations have a valuable opportunity to increase the size, and thus the impact,^15^ of warning labels. Introducing these measures simultaneously would be more efficient for both regulators and tobacco companies, though a combined approach could complicate efforts to delineate the individual effects these policies have on tobacco use.

Third, while many studies have documented how tobacco packaging functions as a marketing medium, few have examined whether re-designing the appearance of tobacco products themselves could promote cessation. For example, changing cigarette sticks from a pristine white colour, which give the impression of purity and may reduce harm perceptions, to an unappealing colour could decrease the psychological distance between tobacco products’ appearance and their effects. Recent studies found that cigarette sticks featuring unattractive colours or graphics were strongly dissuasive,^16^^,^^17^ though these findings require testing within individual jurisdictions to identify optimally dissuasive colours and any unintended effects. Because many tobacco companies already print brand names and other marketing stimuli on cigarette sticks, requiring tobacco products such as sticks or rolling papers to feature dissuasive imagery should be straightforward to implement. Data from Australia, together with findings from studies predicting plain packaging’s likely effects in other countries, suggest this policy will diminish experiences of using tobacco and reinforce the increasingly negative connotations tobacco use evokes.

The strategies outlined above could heighten the mental discomfort that plain packaging will create, particularly given that many tobacco users already report high levels of regret.^18^ Our fourth suggestion is thus that standardized packaging should direct tobacco users to cessation assistance so people experiencing discomfort can resolve these feelings via a supported quit attempt.

While many tobacco packages already provide information about cessation services, these details are often obscurely located and lack visual impact. Complementing plain packaging with highly salient cessation information could channel discomfort tobacco users experience by directing them to expert quitting services. Enhancing the appearance of cessation information could trigger more supported quit attempts, improve quit attempt success rates and enhance the impact plain packaging has on overall tobacco use.^19^ Tobacco companies’ claim^20^ that plain packaging will have no effect on users’ behaviours makes it particularly important to redesign packaging so it foregrounds cessation services, encourages quitting, and facilitates assessment of this policy’s effects.

Studies examining standardized packaging’s effects in Australia now complement evidence from qualitative studies, surveys, naturalistic studies and experimental tasks, and provide robust evidence that standardized packaging of tobacco products reduces smoking prevalence.^8^ Adopting the measures we have outlined here will increase the impact of standardized packaging and subsequently improve public health.
